# Structural Biology of Human H3K9 Methyltransferases

**DOI:** 10.1371/journal.pone.0008570

**Published:** 2010-01-11

**Authors:** Hong Wu, Jinrong Min, Vladimir V. Lunin, Tatiana Antoshenko, Ludmila Dombrovski, Hong Zeng, Abdellah Allali-Hassani, Valérie Campagna-Slater, Masoud Vedadi, Cheryl H. Arrowsmith, Alexander N. Plotnikov, Matthieu Schapira

**Affiliations:** 1 Structural Genomics Consortium, University of Toronto, Toronto, Ontario, Canada; 2 Ontario Cancer Institute and Department of Medical Biophysics, University of Toronto, Toronto, Ontario, Canada; 3 Department of Pharmacology and Toxicology, University of Toronto, Toronto, Ontario, Canada; University of Cambridge, United Kingdom

## Abstract

SET domain methyltransferases deposit methyl marks on specific histone tail lysine residues and play a major role in epigenetic regulation of gene transcription. We solved the structures of the catalytic domains of GLP, G9a, Suv39H2 and PRDM2, four of the eight known human H3K9 methyltransferases in their apo conformation or in complex with the methyl donating cofactor, and peptide substrates. We analyzed the structural determinants for methylation state specificity, and designed a G9a mutant able to tri-methylate H3K9. We show that the I-SET domain acts as a rigid docking platform, while induced-fit of the Post-SET domain is necessary to achieve a catalytically competent conformation. We also propose a model where long-range electrostatics bring enzyme and histone substrate together, while the presence of an arginine upstream of the target lysine is critical for binding and specificity.

**Enhanced version:**

**This article can also be viewed as an enhanced version in which the text of the article is integrated with interactive 3D representations and animated transitions. Please note that a web plugin is required to access this enhanced functionality. Instructions for the installation and use of the web plugin are available in Text S1.**

## Introduction

**Figure 1:**
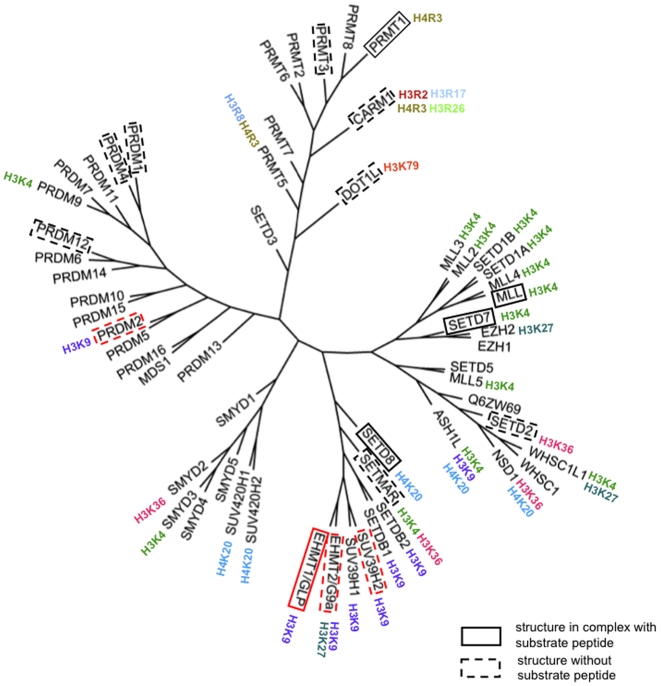
Phylogenetic tree of human histone methyltransferases. Phylogeny is based on a multiple sequence alignment of the methyltransferase domain including the N-SET, Pre_SET, SET, I-SET, and Post-SET motifs. Substrate selectivity was extracted from Kouzarides [1]. Enzymes with solved structure are highlighted by a frame, dotted if no peptide substrate complex is available. Structures solved in the present work are framed in red.

Post-translational modifications of histone proteins regulate chromatin compaction, mediate epigenetic regulation of transcription, and control cellular differentiation in health and disease [1], [2]. Methylation of histone tails is one of the fundamental events of epigenetic signaling [3]. Tri-methylation of lysine 9 of histone 3 (H3K9) mediates chromatin recruitment of HP1, heterochromatin condensation and gene silencing [4], [5]. Similarly, methylation of H3K27 and H4K20 are associated with a repressed state of chromatin, whereas expressed genes are methylated at H3K4, H3K36 and H3K79 ([3], [6] for review). Histone methyltransferases are divided into protein arginine methyltransferases (PRMTs) and histone lysine methyltransferases (HKMTs). HKMTs catalyze the transfer of a methyl group from the co-factor S-adenosyl-L-methionine (SAM) to a substrate lysine and, with the exception of DOT1L, are all organized around a canonical SET domain [7], [8]. The structures of a number of HKMTs have been reported, including ternary complexes of human orthologs with co-factor and substrate peptides (SETD7-H3K4, SETD8-H4K20 and MLL1-H3K4 [9], [10], [11], [12]), as well as *N. crassa* Dim-5 in complex with a H3K9 peptide [13] and a viral protein complexed to H3K27 [14] (Figure 1). These structures collectively highlighted a remarkable plasticity of the peptide-binding site and a lack of clear structural motifs that correlate with sequence selectivity [8], [15].

Methylation of H3K9 in humans relies mostly on members of the Suv39 family, namely EHMT1/GLP, EHMT2/G9a, SUV39H1, SUV39H2, SETDB1 and SETDB2, as well as then non-Suv39 enzymes PRDM2 and ASH1L [6] (Figure 1). Here we report the high-resolution crystal structures of the methyltransferase domains of GLP, G9a, SUV39H2 and PRDM2, and propose a structural mechanism for substrate recognition. Our data also provide important insight to guide the development of potent and selective inhibitors of HKMTs, which are likely to have applications in a variety of diseases including regenerative medicine, oncology and inflammation [16], [17], [18], [19], [20] ([21], [22] for review).

## Results and Discussion

### Overall Structure

**Figure 2:**
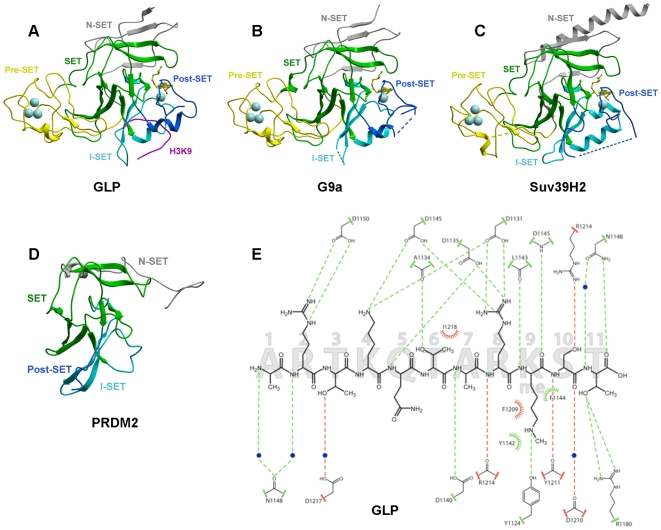
Structures of four human H3K9 HKMTs. (A) a ternary complex of GLP with SAH and a H3K9Me substrate peptide, (B) G9a in complex with SAH, (C) SUV39H2 in complex with SAM and (D) PRDM2, highlighting Pre-Set, SET, I-SET, Post-SET domains and the conserved presence of an N-SET domain. The co-factor is shown as yellow sticks. Residues flanking un-resolved regions are connected by dotted lines. (E) The detail of the interactions between GLP and an H3K9Me peptide.

We have solved the crystal structures of the catalytic domain of four H3K9 methyltransferases: (1) the complexes of GLP/EHMT1 co-crystallized with the co-factor product S-adenosyl-L-homocysteine (SAH) alone (accession code 2IGQ) and with an H3K9me (accession code 3HNA) or H3K9me2 peptide (accession code 2RFI), (2) G9a/EHMT2 (accession code 2O8J) and SUV39H2 (accession code 2R3A) in complex with SAH and SAM respectively, and (3) the non Suv39 protein PRDM2 (accession code 2QPW). The Suv39 structures G9a, GLP and SUV39H2 adopt a typical fold composed of a conserved SET domain and variable I-SET insert, flanked by Pre- and Post-SET regions, and characterized by canonical features such as a pseudo-knot next to the catalytic site, distinct co-factor and substrate binding areas meeting at the site of methyl transfer, and a narrow substrate lysine docking channel [8], [23], [24]. The four structures are also characterized by the presence of an N-SET region located N-terminal to the Pre-SET, that wraps around the core SET domain (Figure 2A–D). The H3K9 peptide lies in a groove formed by the I-SET and Post-SET domains (Figure 2A), as previously observed in other HKMTs ([8] for review), and makes extensive contact with the enzyme through both backbone and side-chain interactions (Figure 2E).

### Methylation State Specificity

**Figure 3:**
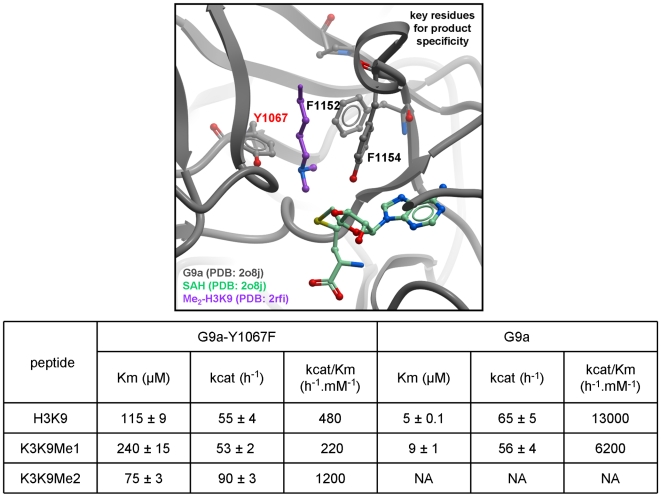
Structural determinants of G9a mono/di-methylation specificity. A model of substrate lysine-bound G9a (Top panel) was generated from the superposition of the active sites of the GPL ternary complex and GLP. Y1067 of G9a stabilizes the di-methylamine end of the substrate lysine in an orientation where the lone-pair is not facing the co-factor, thereby disfavoring transfer of a third methyl group. The Y1067F mutant loses this restriction and can tri-methylate its substrate, as indicated in the table. Previous work had shown that the F1152Y G9a mutant can only mono-methylate H3K9 [29].

Mono-, di- or tri- methylation of H3K9 constitute distinct biochemical signals and are established by distinct histone methyltransferases; G9a and GLP are mono- and di-methylases, and SUV39H2 di- and tri-methylates a mono-methylated substrate. The specificity of PRDM2 is unknown [25]. Several aromatic residues line the GLP channel occupied by the substrate lysine leading to the catalytic site and contribute to methylation state specificity as previously noted for several other HKMTs [9], [12], [26], [27]. Two residues are of particular importance for catalysis. First, a conserved tyrosine residue of the post-SET domain (Y1211 in GLP and Y1154 in G9a) is a major component of the lysine binding channel while its hydroxyl group participates in catalysis. This residue cannot be mutated without losing catalytic activity (Figure 3) [8], [28]. Second, the hydroxyl group of GLP's Y1124 (Y1067 in G9a) hydrogen-bonds to the methyl-accepting nitrogen, thereby inhibiting an orientation of the dimethyl-amine that would favor transfer of a methyl from SAM, as was previously shown for SETD7 ([12], [28] for review). To confirm this model, we showed that, unlike wild-type G9a, the Y1067F mutant is able to tri-methylate H3K9 (Figure 3). Similarly, it was shown that the F1152Y G9a mutant can only mono-methylate H3K9 [29]. Our structures show that this residue is perfectly superimposed with GLP's F1209 (0.1 Å RMSD), which, if mutated to Tyr, would hydrogen-bond with the ε-amine nitrogen of H3K9 and impair the alignment of the accepting amine's lone pair with the methyl-sulfur bond of SAM (Figure 3). Thus, the methylation state selectivity appears to be inversely proportional to the number of tyrosine residues surrounding the methyl accepting nitrogen.

### I-SET Is a Rigid Peptide-Docking Platform

**Figure 4:**
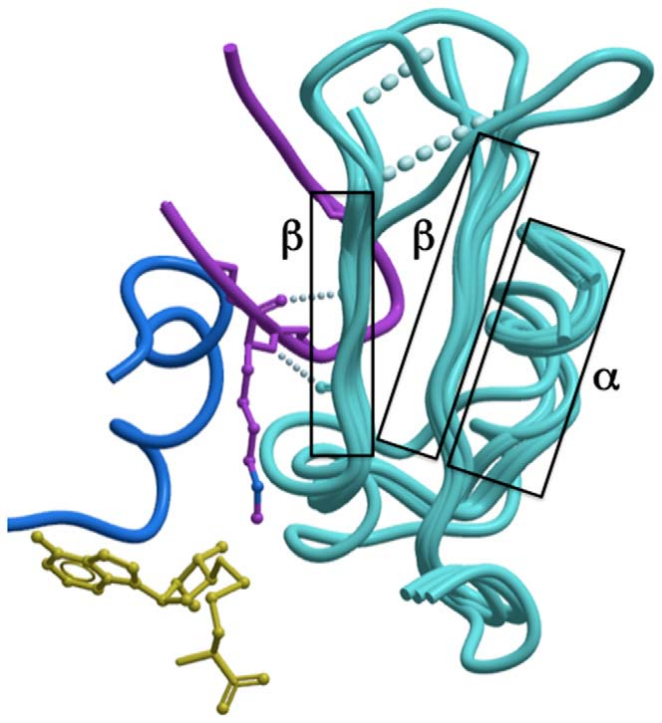
The I-SET domain is relatively rigid and structurally conserved. Structural superimposition of the ternary GLP structure with G9a or Suv39H2 in complex with co-factor and with the apo-structure of PRDM2 shows that the I-SET (cyan) conformation is conserved. The backbone atoms engaged in a double hydrogen-bond with the substrate lysine observed in all available HKMT-peptide complexes are already positioned in the absence of peptide or co-factor.

**Figure 5:**
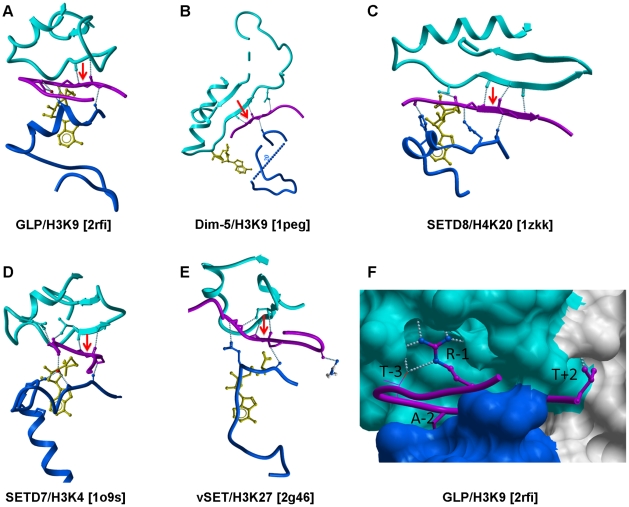
Backbone and side-chain contributions to peptide binding. A–E: Both Post-SET (blue) and I-SET (cyan) backbone atoms are engaged in a network of hydrogen-bonds with the peptide main-chain (magenta). A pair of hydrogen-bonds between backbone atoms of the I-SET and substrate lysine are conserved in all available HKMTs ternary complexes to date (dotted lines flanking red arrow). F: the substrate peptide sits in a groove formed by the I-SET (cyan) and the Post-SET (blue) domains. Peptide side-chains contributing most to the interaction are shown (magenta sticks). The guanidinium group of H3R8 (R-1) makes extensive contacts with the I-SET domain.

The structure of the I-SET domain appears relatively conserved, whether in the apo, co-factor-, or peptide-bound form, and is composed of a helix followed by a two-stranded anti-parallel β-sheet, linked by loops of variable lengths (Figure 4). Superimposition of our six new H3K9 methyltransferase structures (see Materials and Methods) shows that in all cases the first β-strand is in a conformation which would preserve the pair of backbone hydrogen-bonds observed between Lys-9 substrate and strand-1 of I-SET in the GLP-peptide complex (Figure 4). Furthermore, a systematic comparison of the ternary structures presented here for GLP-H3K9 and previously published for Dim-5-H3K9, SETD8-H4K20, SETD7-H3K4, SETD7-TAF10, SETD7-P53, vSET-H3K27 reveals that this pair of hydrogen bonds between the backbone of a single substrate peptide residue and the first strand of I-SET is observed (1) in all HKMT ternary structures to date and (2) always and only at the substrate lysine (Figure 5A–E - SETD7-TAF10 and SETD7-P53 complexes not shown). This suggests that an evolutionary pressure enforces conservation of this “double hydrogen-bond”, which likely plays an important role in the binding mechanism, probably by imposing the proper orientation of the peptide when lysine inserts into the active site; flipping the substrate by 180° in its groove would not allow formation of the double hydrogen bond.

Our structures single-out residue R-1 of H3 (the methylated lysine is used as reference position 0 for peptide residue numbering throughout the text) as the major contributor to the interaction after the substrate lysine itself, with four direct hydrogen-bonds between the arginine guanidinium group and GLP (Figure 2E). This is in agreement with recent mutational analysis showing that no substitution at position -1 is tolerated by G9a [30]. This critical interaction takes place exclusively with I-SET residues (Figure 5F). GLP residues that contribute to substrate binding are conserved in G9a (Figure S1), and it is reasonable to assume that the peptide binding mode observed in GLP holds for G9a. On the other hand, mutation of R-1 to alanine only mildly affects peptide binding to Dim-5, a H3K9 methyltransferase in *N. crassa* [31], indicating that the selectivity mechanism observed for human GLP and G9a is not universal.

**Figure 6:**
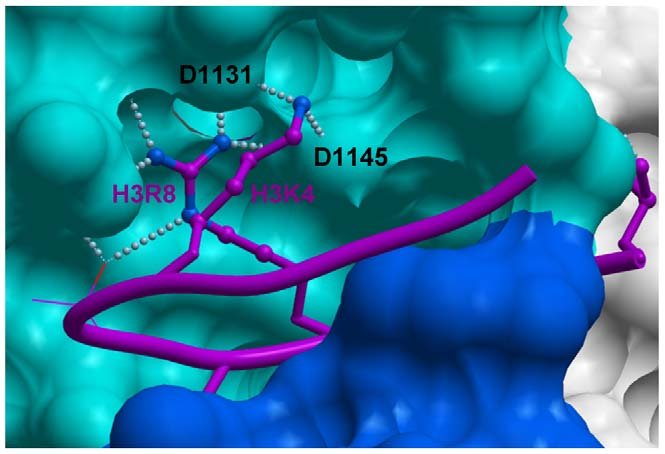
Contribution of H3K4 to H3K9 binding. Our structures of GLP in complex with H3K9me or H3K9me2 show that H3K4 folds on top of H3R8, making polar interactions with D1131 and D1145 of GLP.

Intriguingly, our structures of GLP in complex with mono- or di-methylated susbtrate peptides reveal that residue H3K4 is making 2 hydrogen-bonds with the D1131 and D1145 side-chains of the I-SET domain (Figure 6), which suggests that H3K4 methylation may lower binding affinity, and reduce H3K9 methylation efficiency. We tested this hypothesis, and observed a mild decrease of 43% in affinity of GLP for a H3K9 peptide trimethylated at lysine 4, and similar reduction in enzymatic efficiency, while Kcat was unaffected. Mono and di-methylation of H3K4 had no or very limited effect (data not shown). It is not clear whether this variation is biologically significant.

These results suggest a model in which a mostly pre-formed I-SET domain acts as a receiving platform for the histone 3 tail. Binding includes a conserved pair of hydrogen-bonds with the backbone of the substrate lysine, and critical contacts with a basic side-chain upstream of the methyl acceptor.

### Mobile Post-SET Domain Closes onto the Peptide Substrate

**Figure 7:**
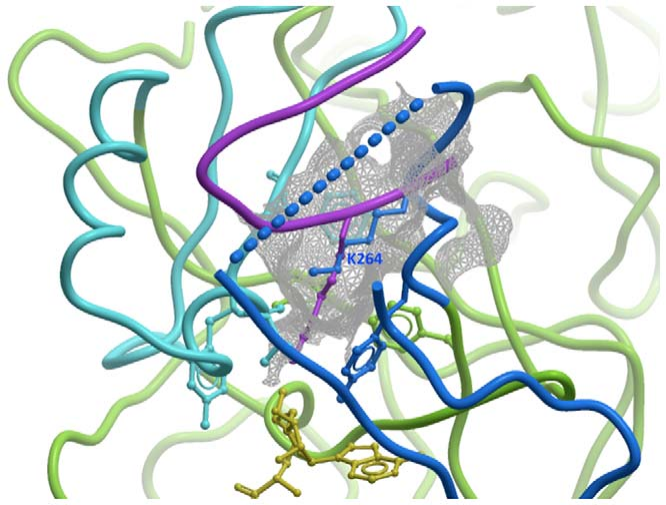
Auto-inhibitory conformation of SUV39H2. In our structure of SUV39H2, the C-terminus of the Post-Set domain (blue) adopts a conformation that positions its K264 side-chain (blue sticks) half-way into the substrate lysine channel (gray mesh). The H3K9me peptide (magenta) from a superimposed GLP-H3K9me structure is shown as a reference. SET and Post-SET of SUV39H2 are colored green and blue respectively.

The Post-SET domains of G9a, GLP, SUV39H2, but not PRDM2 include a ZnCys motif previously observed in the structures of Dim-5 [13] and the H3K4 methyltransferase MLL1 [10]. The Post-SET domains of G9a and GLP present an α-helix that contributes to peptide binding where other HKMTs have a loop. Unlike I-SET, Post-SET is absent from the PRDM2 structure, which lacks co-crystallized SAM or SAH (Figure 2D). It is partially folded in the structures of G9a and SUV39H2 in complex with SAH and SAM respectively (Figure 2B–C), and fully ordered in the ternary complexes of GLP with SAH and H3K9 peptide (Figure 2A). As previously observed with other HKMTs ([9], [11], [8] for review), the co-factor contributes to the formation of a hydrophobic, mostly aromatic cluster (composed of Post-SET Y1211/Y1154/Y261, F1215/F1158, W1216/W1159/L298, F1223/F1166/T285 and SET H1170/H1113/H220 in GLP/G9a/SUV39H2) necessary for partial folding of the Post-SET domain. Surprisingly, in our structure of SUV39H2, Post-SET Lys-264 is inserted into the partially formed substrate lysine binding channel, which may represent some form of auto-inhibitory mechanism (Figure 7). Post-SET is fully structured only when bound to the substrate peptide (Figure 2A), or to a small molecule inhibitor [32], but the density is incomplete otherwise (Figure 2B–D). This implies that Post-SET is naturally flexible, which may be important for peptide turn-over, as recently proposed [10].

Altogether, these results suggest the presence of three conformational states for the Post-SET domain. (1) A flexible or even disordered state when no co-factor or peptide is bound. (2) A loose conformation when SAM or SAH, but no peptide is bound, which may also accommodate non-specific sequences. (3) A more rigid conformation in which the I-SET domain closes onto the substrate peptide. These states are probably in a dynamic equilibrium, with the co-factor and substrate shifting the equilibrium toward conformation 3.

### Long Range Electrostatics Attract Histone Peptides to the HKMT Binding Groove

**Figure 8:**
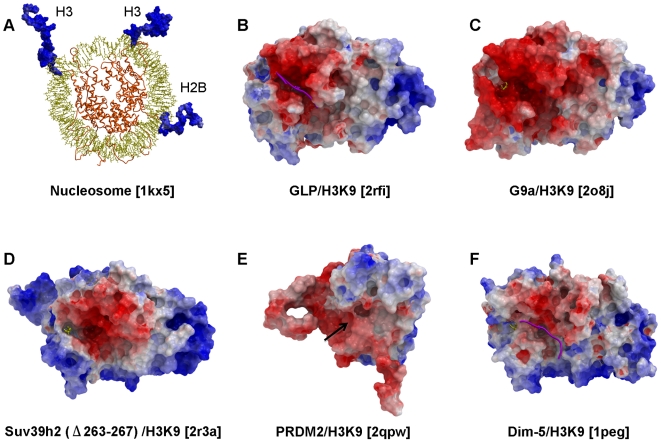
Electrostatic component to H3K9 peptide binding. While the overall electrostatic profile of available H3K9 methyltransferases structures varies, the peptide-binding groove is consistently electronegative (B–E: this work, F: *N. crassa* methyltransferase Dim-5), in contrast with the largely positive electrostatic potential of histone tails (A). When present, the substrate peptide is shown in magenta. Residues 264–267 of SUV39H2 were partially occupying the binding site and were removed. The Post-SET domain of PRDM2 is entirely disordered and the position of the substrate lysine binding channel is indicated with a black arrow.

Histone tails are rich in lysines and arginines and consequently are electropositive (Figure 8A). Mapping the electrostatic potential along the molecular surface of GLP, G9a, SUV39H2 and PRDM2 shows that the peptide-binding groove is consistently electronegative (Figure 8B–E). This feature is also conserved in the structure of the N. crassa H3K9 methyltransferase Dim-5 (Figure 8F). This suggests that non-specific long-range electrostatic attractions play an evolutionarily conserved role in guiding the substrate-binding groove towards histone tails.

Based on the four HKMT structures presented here, we propose a mechanism for selective lysine H3K9 methylation, in which (1) long-range electrostatics attract the enzyme onto basic histone tails, (2) a pre-formed I-SET domain carries structural determinants necessary for specific interactions with the substrate peptide, and (3) catalytically competent conformation is achieved by subsequent closing of the Post-SET domain on the substrate. Considering the electronegative potential of the binding groove, our analysis suggests that HKMT inhibitors should be rather basic. To achieve selectivity, inhibitors should bind sites with clear interaction field potential occupied by residues distal to the substrate lysine. A recent co-crystal structure of the first specific HKMT inhibitor supports these general concepts [32].

## Materials and Methods

### Cloning, Expression and Purification of Human GLP

DNA fragments encoding the methyltransferase domain of human GLP (residues 951-1235), G9a (residues 913–1193), SUV39H2 (residues 112–410) and PRDM2 (residues 2–148) were amplified by PCR and sub-cloned into the pET28a-LIC vector (http://www.sgc.utoronto.ca/SGC-WebPages/toronto-vectors.php), downstream of the poly-histidine coding region. The methyltransferase domains of the above proteins were expressed in *E.coli* BL21 (DE3) codon plus RIL strain (Stratagene) by addition of 1 mM isopropyl-1-thio-D-galactopyranoside and incubated overnight at 15°C. The proteins were purified as following: harvested cells were resuspended in phosphate-buffered-saline (pH 7.4) supplemented with 250 mM NaCl, 2 mM β-mercaptoethanol, 5% glycerol, 0.1% Igepal and 1 mM phenylmethyl sulfonyl fluoride. The cells were lysed by passing through a microfluidizer (Microfluidics Corp.). The lysate was loaded onto a 5 ml HiTrap Chelating column (GE Health Care), charged with Ni^2+^. The column was washed with 10 column volume of 20 mM Tris-HCl (pH 8.0), containing 250 mM NaCl and 50 mM imidazole, 5% glycerol, and the protein was eluted with elution buffer (20 mM Tris-HCl, pH 8.0, 250 mM NaCl, 250 mM imidazole, 5% glycerol). The protein was loaded onto a Superdex 200 column (GE Health Care) equilibrated with 20 mM Tris-HCl (pH 8.0) and 150 mM NaCl. Thrombin (Sigma) was added to combined fractions containing the target proteins to remove the His_6_- tag. The protein was further purified to homogeneity by ion-exchange chromatography.

### Crystallization

Purified GLP, G9a and SUV39H2 proteins were crystallized in the presence of S-adenosyl-L-homocysteine (Sigma) or S-adenosyl-L-methionine (Sigma) using the hanging drop vapor diffusion method at 20°C by mixing equal volume of the protein solution with the reservoir solution. The GLP-SAH binary complex (protein/SAH molar ratio of 1∶5) was crystallized in 20% PEG 4,000, 10% isopropanol, 0.1 M HEPES (pH 7.5). The G9a-SAH complex (protein/SAH molar ratio of 1∶5) was crystallized in 20% PEG 3,350, 0.2 M NaF, 0.1 M Bis-Tris propane (pH 7.5), 5% ethylene glycol. The SUV39H2-SAM complex (protein/SAM molar ratio of 1∶10) was crystallized in 20% PEG 10, 000, 0.1 M HEPES (pH 7.5). The GLP-SAH-H3K9me and GLP-SAH-H3K9me2 complexes (protein/SAH/peptide molar ratio of 1∶5∶10) were crystallized in 16% PEG 4,000, 10% isopropanol, 0.1 M HEPES (pH 7.5). Purified PRDM2 was crystallized using hanging drop vapor diffusion method at 20°C by mixing 1.5 µl of the protein solution with 1.5 µl of the reservoir solution containing 22% PEG 5,000 MME, 0.2 M ammonium sulfate, 0.1 M MES (pH 7.0). All the crystals were soaked in the corresponding mother liquor supplemented with 20% glycerol as cryoprotectant before freezing in liquid nitrogen, except the G9a-SAH complex crystals, for which paraton-N was used as cryoprotectant.

### Data Collection and Structure Determination

X-ray diffraction data were collected at 100 K at beamline 17ID of Advanced Photon Source (APS) at Argonne National Laboratory, beamline X25 of the National Synchrotron Light Source, beamline A1 of Cornell High Energy Synchrotron Source (CHESS), Cornell University, and a Rigaku FR-E home source. Data were processed using the HKL-2000 software suite [33]. The structures of methyltransferase domain of GLP, G9a, and SUV39H2 were solved by molecular replacement using the program MOLREP [34]. ARP/wARP [35] was used for automatic model building. Graphics program COOT [36] was used for model building and visualization. PRDM2 structure was solved by single-wavelength anomalous diffraction (SAD) at low resolution, using a seleno-methionine derivative crystal with the program SHELXD [37], and the phasing was performed using SHELXE [38]. The low resolution structure was used as model to solve the native structure at higher resolution. Crystal diffraction data and refinement statistics for the structure are displayed in Table 1. One residue is in a disallowed area of the Ramachandran plot in our GLP (M1049) and G9a (I992) structures. This strained residue maps at a conserved location, remote from the peptide and cofactor binding sites.

**Table 1:** Summary of X-ray diffraction data.

	EHMT1+AdoHcy	EHMT1+AdoHcy+H3K9me2	EHMT1+AdoHcy+H3K9me	EHMT2+AdoHcy	SUV39H2+AdoMet	PRDM2
PDB Code	2IGQ	2RFI	3HNA	2O8J	2R3A	2QPW
Data collection						
X-ray source	NSLS-X25	APS-17ID	APS-17ID	APS-17ID	Rigaku FR-E	CHESS-A1
Space group	P2_1_ 2_1_ 2_1_	P2_1_ 2_1_ 2_1_	P2_1_ 2_1_ 2_1_	P1	P1 2_1_ 1	P2_1_ 2_1_ 2_1_
Cell dimensions						
*a*, *b*, *c* (Å)	74.52, 95.86, 102.12	84.59, 85.63, 95.67	83.53, 83.37, 95.13	56.59, 70.96, 83.05	45.75, 63.92, 64.53	36.46, 51.20, 70.89
*α*, *β*, *γ* (°)	90.00, 90.00, 90.00	90.00, 90.00, 90.00	90.00, 90.00, 90.00	81.11, 75.13, 88.39	90.00, 109.33, 90.00	90.00, 90.00, 90.00
Resolution (Å) (highest resolution shell)	69.84-2.00 (2.07-2.00)	42.80-1.60 (1.64-1.60)	62.75-1.50(1.58-1.50)	79.30-1.80 (1.86-1.80)	50.00-2.00 (2.08-2.00)	50.00-1.79 (1.86-1.79)
Unique reflections	44160	89676	106827	100323	22956	23999
*R*_sym_ (%)	9.6	8.0	9.8	4.8	8.4	6.3
*I*/σ*I*	8.8(1.3)	10.8 (1.4)	15.2(4.3)	13.7(3.0)	15.0(3.4)	34.3 (6.0)
Completeness (%)	92.6(83.2)	96.5 (67.2)	100.0(100.0)	92.3(72.7)	96.6 (92.9)	99.7 (99.3)
Redundancy	3.7(2.0)	7.4 (3.0)	8.2(8.2)	3.2(3.2)	2.4 (2.3)	7.2 (6.4)
**Refinement**						
Resolution (Å)	42.11-2.00	42.80-1.59	62.75-1.50	79.31-1.80	35.38-2.00	41.49-1.79
No. reflections (test set)	44160 (2365)	85102 (4475)	101405 (5324)	100323 (5287)	21763 (1182)	13205 (841)
*R*_work/_ *R*_free_ (%)	19.1/23.7	19.4/22.0	16.7/19.3	18.4/22.1	17.9/21.3	17.1/22.2
No. atoms						
Protein	4084	4322	4550	8331	2154	1192
Cofactor	60	60	60	120	31	N/A
Water	566	681	888	1780	254	145
B-factors (Å^2^)						
Protein	31.7	24.8	15.1	24.2	29.2	21.8
Cofactor	44.5	33.2	13.1	24.3	25.7	N/A
Water	40.4	32.6	31.9	32.8	38.3	36.2
R.m.s deviations						
Bond lengths (Å)	0.015	0.009	0.009	0.013	0.017	0.019
Bond angles (°)	1.53	1.17	1.28	1.35	1.55	1.51
Ramachandran plot % residues						
Favored	82.9	85.0	85.1	86.4	86.7	87.8
Additionally allowed	16.2	14.1	14.3	13.2	13.3	12.2
Generously allowed	0.5	0.4	0.2	0	0	0
Disallowed	0.5	0.4	0.4	0.4	0	0

### Histone Methyltransferase Assay

The SAHH-coupled assay described by Collazo et al. [39] was optimized and employed to assay the activity of G9a. This assay utilizes S-adenosylhomocysteine hydrolase (SAHH) to hydrolyze the methyltransfer product S-adenosylhomocysteine to homocysteine and adenosine in the presence of adenosine deaminase which converts adenosine to inosine. The homocysteine concentration is then determined through conjugation of its free sulfhydryl moiety to a thiol-sensitive fluorophore, ThioGlo (Calbiochem). Assays were performed at room temperature in 25 mM potassium phosphate buffer pH 8, 1 mM EDTA, 2 mM MgCl_2_ and 0.01% Tween 20. Series of control experiments were conducted to establish the optimum assay condition for each methyltransferase and the optimum conditions were used to determine the kinetic parameters for GLP. Assay cocktails were prepared with 5 µM SAHH to avoid any SAH accumulation while produced from the methyltransferase reaction, 3 U/ml of adenosine deaminase from Sigma, 70 µM SAM, and GLP. The peptide concentrations were varied over the range of 2 µM to 4 mM. Assays were initiated by the addition of peptide and immediately after starting the reaction, 2x volume of 20 µM ThioGlo solution was added to each well. The methylation reaction was followed by monitoring the increase in fluorescence using Biotek Synergy2 plate reader with 360/40 nm excitation filter and 528/20 nm emission filter for 20 min in 384 well-plate format. Homocysteine generated in the assay was quantified using standard curves. Activity values were corrected by subtracting background caused by the peptide or the protein. K_m_ and k_cat_ values were calculated using the Michaelis-Menten equation and Sigmaplot 9.0. Standard deviations were calculated from two independent experiments.

### Structure Superimposition and Electrostatic Potential Coloring

Optimal structure superimpositions were identified with ICM (Molsoft LLC). Briefly, the algorithm uses an iterative procedure to find the best “alignable” main chain core in both structures based on seed alignments of 15 residues as follows: (1) start with the most reliable seed alignment of 15 residues; (2) set all weights to 1; (3) perform weighted superposition and evaluate RMSD; (4) calculate the deviation Di for each backbone atom pair; (5) sort the deviations and find the deviation D50 corresponding to 50-percentile of the deviation array; (6) calculate weights W according to the formula Wi = exp(-D50^2^/Di^2^); (7) go back to step 3 unless a limit of 10 iterations is reached. The electrostatic potential was calculated with ICM using a boundary element solution of the Poisson equation. Color saturation was set to calculated values of +/− 5 kcal/electron units (+5 = blue −5 = red) when the electrostatic potential was projected on molecular surfaces.

## Supporting Information

Figure S1 Sequence alignment of the methyltransferase domain of H3K9 HKMTs. Residues within 4Å of bound H3K9 peptide in our GLP-H3K9me complex are highlighted in red. The three aspartate making polar interactions with arginine H3R8 are colored blue. The sequence of PRDM2 is too divergent from other H3K9 HKMTs and was not included in the alignment. Large inserts present in SETDB1 and SETDB2 sequences are not shown for clarity.
(1.11 MB TIF)

Datapack S1 Standalone iSee datapack - contains the enhanced version of this article for use offline. This file can be opened using free software available for download at http://www.molsoft.com/icm_browser.html.
(ICB)

Text S1 Instructions for installation and use of the required web plugin (to access the online enhanced version of this article).
(0.75 MB PDF)
