# Supplementary figures and images for: Structural Biology of Human H3K9 Methyltransferases
Source: PLoS One. 2010 Jan 11;5(1):e8570. doi: 10.1371/journal.pone.0008570 (PMC2797608; doi:10.1371/journal.pone.0008570)

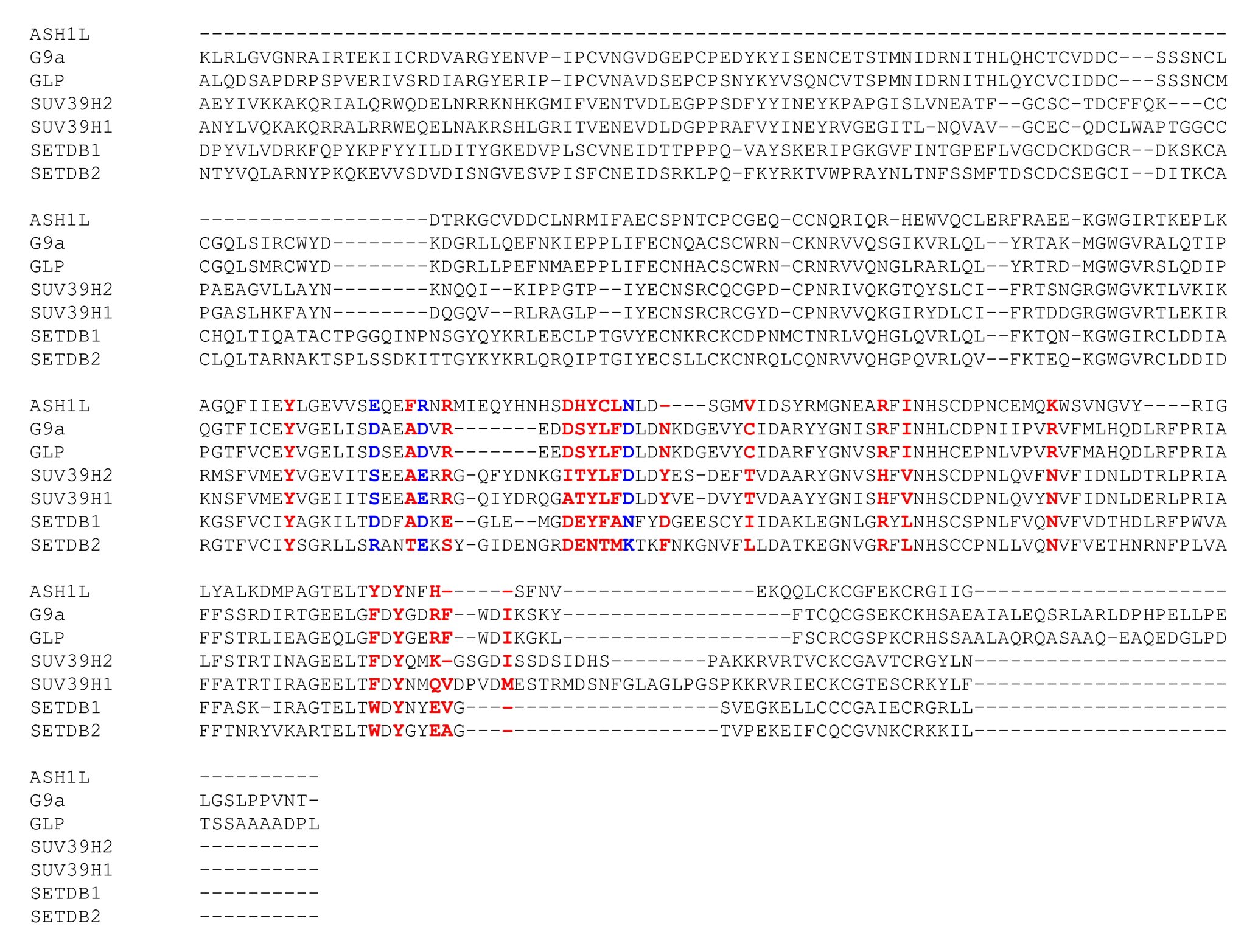

Supplement: Figure S1 — Sequence alignment of the methyltransferase domain of H3K9 HKMTs. Residues within 4Å of bound H3K9 peptide in our GLP-H3K9me complex are highlighted in red. The three aspartate making polar interactions with arginine H3R8 are colored blue. The sequence of PRDM2 is too divergent from other H3K9 HKMTs and was not included in the alignment. Large inserts present in SETDB1 and SETDB2 sequences are not shown for clarity. (1.11 MB TIF) [file pone.0008570.s001.tif]
